# Prophylactic negative-pressure wound therapy for prevention of surgical site infection in abdominal surgery: a nationwide cross-sectional survey

**DOI:** 10.1007/s13304-021-01017-3

**Published:** 2021-04-10

**Authors:** Elin Roos, Jonathan Douissard, Ziad Abbassi, Nicolas C. Buchs, Christian Toso, Frédéric Ris, Jeremy Meyer

**Affiliations:** 1 Department of Global Public Health, Karolinska Institutet, 171 77 Stockholm, Sweden; 2 Division of Digestive Surgery, University Hospitals of Geneva, Rue Gabrielle-Perret-Gentil 4, 1211 Geneva 14, Switzerland; 3 Medical School, University of Geneva, 1205 Genève, Switzerland; 4 County Council of Östergötland, Linköping University, Linköping, Sweden

**Keywords:** Negative therapy, Negative-pressure therapy, PREVENA, PICO

## Abstract

Our objective was to determine current practice in Switzerland regarding the use of pNPWT in abdominal surgery. An online survey was carried out to evaluate the use of pNPWT among abdominal surgeons in Switzerland. One hundred and ten participants replied to the survey from 16.12.2019 to 15.01.2020. Eleven were excluded, leaving 99 responders for analysis. Seventy participants (70.7%) were using pNPWT, 3 (3%) have stopped using it and 26 (26.3%) have never used it. pNPWT was used on midline laparotomy by 63 responders (90%), closed stoma wounds by 21 (30%), closed perineal wounds by 20 (28.6%), Pfannenstiel incisions by 18 (23.7%), groin incisions by 16 (22.9%), subcostal incisions by 13 (18.6%), Mc Burney incisions by 3 (4.3%) and other incisions by 18 (25.7%). Forty-eight participants (68.6%) used pNPWT on less than 10% of patients, 14 (20%) on 10–25% of patients, six (8.6%) on 25–50% of patients and two (2.9%) on 75–100% of patients. Suggestions for improvement to pNPWT were: better sealing, recyclable system, better adaptation to the perineum, smaller device, reduced cost and possibility to check the surgical wound through the dressing. In conclusion, pNPWT is widely used among Swiss surgeons, mostly on midline incisions. However, most of them apply pNPWT on a small proportion of patients only. Suggestions for improvement were a better sealing for complex wounds, reduced cost and possibility to check the wound during the therapy.

**Supplementary Information:**

The online version contains supplementary material available at 10.1007/s13304-021-01017-3.

## Introduction

Prophylactic negative-pressure therapy (pNPWT) consists of an aspirative plaster connected to a pump applying negative pressure on a closed surgical wound. In this regards, pNPWT differs from the usual negative-pressure wound therapy applied on an open wound for secondary healing.

pNPWT was documented to lower the incidence of surgical site infection (SSI) and wound dehiscence in closed laparotomy incisions [1–3]. Notably, we demonstrated that pNPWT allowed to decrease the incidence of SSI by 12 percentage points after laparotomy [4].

The financial burden of surgical site infection (SSI) is important and efforts should be made to reduce the incidence of SSI [5]. However, experience from our team shows that pNPWT has not gained widespread popularity among surgeons in Switzerland and abroad, notably due to the lack of RCT-supported evidence [6] and the cost of the technique.

Therefore, our objective was to determine the proportion of abdominal surgeons using pNPWT in their current practice, the indications they consider for pNPWT, the types of commercial devices used, the postulated limitations of the techniques and suggestions for improvement.

## Materials and methods

### Type of study

The study consisted in an online survey developed on the software Evalandgo (Pro edition) [7], which was carried out among members of the Swiss Surgical Society. The first invitation to the survey was sent on the 16.12.2019 and a reminder was sent on the 13.01.2020. The survey was closed on the 15.01.2020. The study did not require ethical clearance.

### Population

The link to the online form was sent to members of the Swiss Surgical Society. Answers were retained only if all questions were answered and if the member were specialists in surgery. Students and/or residents without completed surgical qualification were excluded from analysis.

### Variables of interest

The survey contained 22 questions, with both quantitative and qualitative aspects (Table S1). Briefly, participants were asked about their surgical qualifications, when they learned about pNPWT, the type of pNPWT devices available and used in their hospitals, the proportions of patients undergoing abdominal surgery who benefited from pNPWT, the types of wounds on which the participants applied pNPWT, the indications for pNPWT (in terms of risk factors for SSI), and their personal opinions about the eventual issues encountered with pNPWT and propositions for improvement.

### Statistical analysis

Results were expressed as *n* (%) for categorical variables and as means (± SEM) for continuous variables. Descriptive analysis was performed using the PRISM software (GraphPad, version 5).

## Results

### Participants

Nine hundred and seven surgeons have been solicited for the survey by the Swiss Surgical Society. One hundred and ten replied to the survey. Eleven participants were excluded; 5 for not being specialists in surgery and 6 for not completing the survey, leaving 99 patients for analysis. In terms of gender, 81 participants (81.8%) were males of 18 were females (18.2%). The mean age was 49.8 ± 1 years. Ninety-nine participants (100%) were specialists in general surgery, 41 (41.4%) were also specialists in visceral surgery and 14 (14.1%) had a European board certification for a subspecialty in surgery. The mean number of years of experience after obtaining the Swiss qualification for general surgery was 16.4 ± 1.1 years. The main field of surgical practice were general surgery for 42 (42.4%) participants, upper gastrointestinal surgery for 5 (5.1%), lower gastrointestinal surgery for 27 (27.3%), hepatobiliary surgery for 4 (4%), transplantation surgery for 2 (2%) and others for 19 (19.2%). Twenty-nine (29.3%) participants were working in a University Hospital, 49 (49.5%) in a regional hospital and 21 (21.2%) in a private clinic.

### Use of pNPWT

**Fig. 1:**
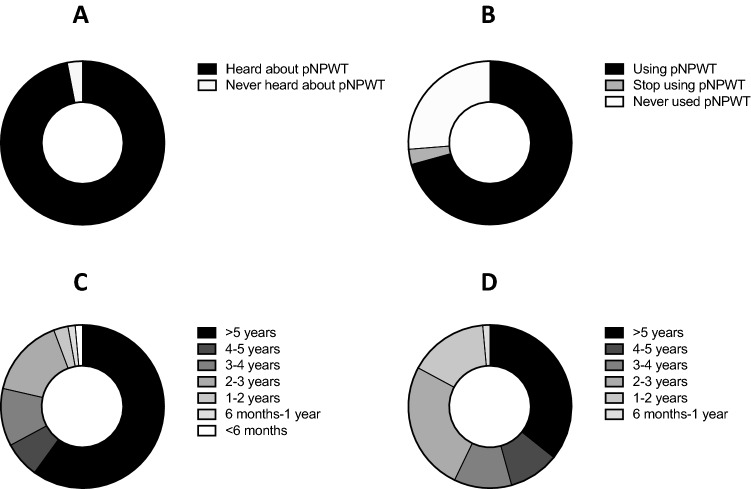
Use of pNPWT among Swiss surgeons practising abdominal surgery. **a** Information regarding pNPWT among Swiss surgeons. **b** Use of pNPWT among Swiss surgeons in abdominal surgery. **c** Time when pNPWT was made available in Swiss surgeons’ institutions. **d** Time when Swiss surgeons have first used pNPWT in abdominal surgery

Ninety-six (97%) participants have heard about pNPWT and three (3%) were not informed about the possibilities of pNPWT (Fig. 1a). Seventy participants (70.7%) reported using pNPWT, 3 (3%) had stopped using it and 26 (26.3%) have never used it (Fig. 1b).

Main reason for discontinuation of use was lack of observed effect, notably on the post-operative incidence of SSI.

For those using pNPWT (70 participants), 47 participants (60%) had heard about pNPWT more than 5 years ago, 26 (37.1%) between 1 and 5 years and 2 (2.9%) less than 1 year ago (Fig. 1c). pNPWT was made available in participants’ institutions for more than 5 years for 25 participants (35.7%), more than 4 years ago for 7 (10%), more than 3 years ago for 8 (11.4%), more than 2 years ago for 18 (25.7%), more than 1 year ago for 11 (15.7%) and more than 6 months ago for 1 (1.4%) (Fig. 1d).

### Types of devices

**Fig. 2:**
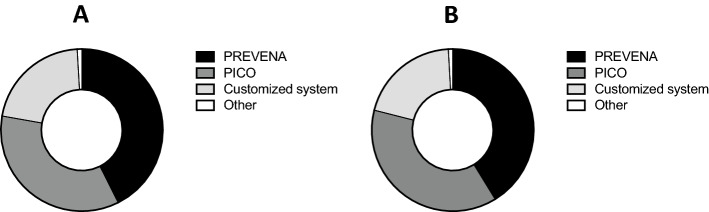
Types of pNPWT systems used by Swiss surgeons for abdominal surgery. **a** pNPWT systems available for Swiss surgeons in their institutions. **b** pNPWT systems used by Swiss surgeons

Among participants using pNPWT, 46 (65.7%) had access to the PREVENA incision management system (KCI, Acelity, San Antonio, USA), 38 (54.3%) to the PICO single use negative pressure wound therapy system (Smith & Nephew, Hertfordshire, UK), 23 participants (32.9%) performed pNPWT using a customized system from usual NPWT (V.A.C.^R^ system, KCI, Acelity, San Antonio, USA) and one (1.4%) participant used another system (Fig. 2a). The other reported systems was the MEDELA system (Medela AG, Baar, Switzerland). During their surgical practice, 47 (67.1%) participants have used the PREVENA incision management system, 43 (61.4%) the PICO single use negative pressure wound therapy system, 23 (32.9%) a customized system from usual NPWT and 1 (1.4%) another system (Fig. 2b).

### Indications for pNPWT

**Fig. 3:**
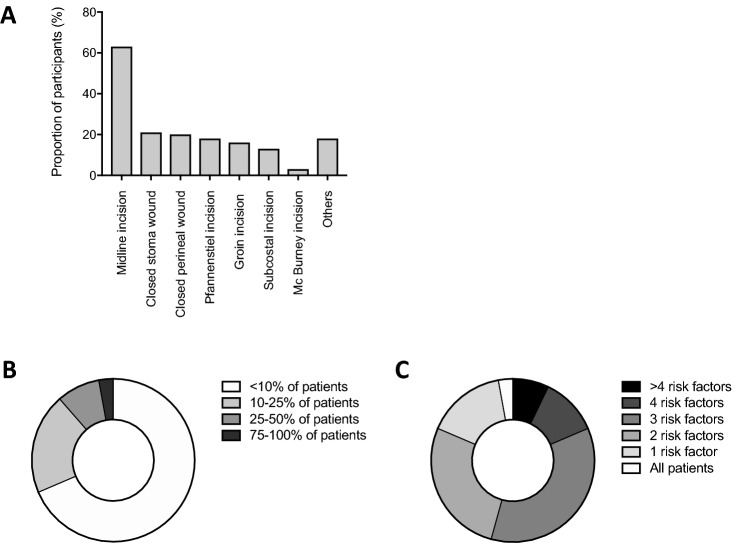
Indications for pNPWT among Swiss surgeons for abdominal surgery. **a** Types of wounds on which Swiss surgeons applied pNPWT. **b** Proportions of patients on which Swiss surgeons applied pNPWT. **c** Minimal number of risk factors for wound-related complications considered by Swiss surgeons to be an indication for pNPWT

pNPWT was used on midline laparotomy by 63 participants (90%), closed stoma wounds (after stoma closure) by 21 (30%), closed perineal wounds by 20 (28.6%), Pfannenstiel incisions by 18 (25.7%), groin incisions by 16 (22.9%), subcostal incisions by 13 (18.6%), Mc Burney incisions by 3 (4.3%) and other incisions by 18 (25.7%) (Fig. 3a). Forty-eight participants (68.6%) used pNPWT on less than 10% of patients, 14 (20%) on 10–25% of patients, 6 (8.6%) on 25–50% of patients and 2 (2.9%) on 75–100% of patients (Fig. 3b). Five participants (7.1%) considered that patients should have > 4 risk factors for SSI to benefit from pNPWT, 8 (11.4%) 4 risk factors, 25 (35.7%) 3 risk factors, 19 (27.1%) 2 risk factors, 11 (15.7%) 1 risk factor and 2 (2.9%) applied it on all patients (0 risk factor) (Fig. 3c). Risk factors were defined as: emergency laparotomy, colorectal surgery, diabetes, obesity, thickness of subcutaneous tissue, contaminated wound and immunosuppression (Table S1).

### Personal opinions

pNPWT was considered as efficient in preventing SSI for 60 (85.7%) participants, wound dehiscence for 41 (58.6%), incisional hernia for 14 (20%) and bad aesthetic aspect of the scar for 3 (4.3%). Four (5.7%) participants did not think that pNPWT was efficient in preventing any of these wound-related complications. The most efficient system was considered by the participants to be the PREVENA incision management system by 31 participants (44.3%), the PICO single use negative pressure wound therapy system by 20 (28.6%), a customized system from usual NPWT by 14 (20%) and another system by 3 (4.3%). The major limitations of pNPWT reported by participants were the cost of the therapy, the difficulties encountered when applying pNPWT on particular areas, such as the perineum or in close proximity to drains and/or stomas, and also lack of patient’s mobility due to the device. Of note, some participants reported the need for more evidence regarding the effect of pNPWT in preventing wound-related complications in abdominal surgery. As a consequence, suggestions for improvement to pNPWT were: better sealing, recyclable system, better adaptation to the perineum, smaller device, reduced cost and possibility to check the surgical wound through the dressing.

## Discussion

In the present study, we showed that the majority of Swiss surgeons (97%) practicing abdominal surgery knew about pNPWT for prevention of wound-related complications after open abdominal surgery. pNPWT was widely available in participants’ hospitals, with the most common commercial devices thoroughly represented. However, 26.3% of participants have reported to have never used pNPWT in abdominal surgery. We note that the literature showing a beneficial effect of pNPWT in abdominal surgery on the incidence of SSI is recent [2–4] and time for adoption of the technique might be required. The availability of the systems in participants’ hospitals might therefore be the consequence of earlier adoption of the technique by other specialties, such as orthopedics.

Moreover, three participants stopped using pNPWT due to postulated lack of observed effect on the prevention of wound-related complications. This raises the question whether this lack of observed effect is merely due to an accumulation of unlikely events (the beneficial effect of pNPWT being proven by meta-analyses [1, 2, 4] and the technique being recommended by the National Institute for Health and Care Excellence (NICE) [8]), or the fact that pNPWT might be less efficient in sub populations of patients with less risk factors for wound-related complications or with more difficult application of pNPWT (for example close to drains and/or stomas and/or on the perineum). Further, the possibility of a potential selection bias, pNPWT being applied by some surgeons only in patients with higher risk factors for SSI, might be evoked.

We note that 21.3% of participants were performing pNPWT using a device they customize from the usual V.A.C. system for secondary healing, applying it on a closed skin. It would be interesting to compare the usual commercial pNPWT systems in terms of prevention of wound-related complications, but also to determine which system is the most efficient in terms of cost–benefit ratio. Indeed, cost of the therapy was one of the subjective limitations mentioned by participants. On this aspect, to our knowledge, no study specifically performed an economical analysis of pNPWT in abdominal surgery. We can imagine that the conclusion of such an analysis would be impacted by the cost of the devices assessed, as well as the number of patients needed to treat to avoid one case of surgical-wound related complication. As a corollary, a better definition of the patients for which pNPWT would be the most efficient is desirable, as it would allow to refine the indications for pNPWT in abdominal surgery. Further, assessing the cost–benefit ratio of the technique is important for low income countries, where cost of the therapy constitutes a significant limitation for its adoption [9].

In the present survey, we showed that participants used pNPWT on an important diversity of abdominal wounds. Of note, the pooled literature showing an effect of pNPWT reflects that diversity [2]. Further, new indications have emerged, such as perineal wounds after abdomino-perineal resection [10–17], which carry a high incidence of post-operative wound complications [18–20]. The effect of pNPWT might be consequent in that subpopulation of patients and deserves further investigation. We think that the effect of pNPWT on prevention of wound-related complications should be pondered according to the type of wounds, which also impact on the risk of wound-related complications [4, 21]. Further, most participants only used pNPWT on a small proportions of patients (68.57% used pNPWT in less than 10% of patients). Once again, this shows that participants performed a selection of patients, probably according to the risk of wound-related complications, and applied pNPWT only in high-risk patients. For instance, 81.42% of participants declared applying pNPWT on patients with two and more risk factors for SSI. We believe that a better definition of patients who would benefit from pNPWT, providing the number needed to treat to avoid one case of wound-related complication, as well as a cost–benefit analysis of that population, might allow a better adoption of pNPWT and lead to substantial savings for healthcare systems. Noteworthy, we showed in a meta-analysis that the effect of pNPWT on the prevention of SSI was more pronounced in studies with an incidence of SSI ≥ 20% in the control arm [4].

Further, studies in the field usually restricted investigations on the effect of pNPWT on prevention of SSI. We think that long-term wound outcomes should be investigated, as they might alter the results of a cost–benefit analysis of pNPWT. For instance, SSI constitutes an important risk factor for incisional hernia [22], whose constitutes a costly complication [23]. Preventing SSI using pNPWT might also allow to prevent incisional hernia, and therefore reduce the cost of wound-related complication for healthcare systems.

To conclude, pNPWT for prevention of wound-related complications is widely used among Swiss surgeons, mostly on abdominal midline incisions. However, most of them apply pNPWT on a small proportion of patients only, selected based on risk factors for wound-related complications. Suggestions for improvements were a better sealing for complex wounds and possibility to check the wound during the therapy. Further studies are required for better implementation of the technique, selection of the patients who might benefit from it and precise evaluation of the benefits for healthcare systems.

## Supplementary Information

Below is the link to the electronic supplementary material.

Supplementary file1 (DOCX 23 KB)

## Abbreviations

pNPWT: Prophylactic negative-pressure wound therapy
SSI: Surgical site infection
