# Supplementary material for: Prophylactic negative-pressure wound therapy for prevention of surgical site infection in abdominal surgery: a nationwide cross-sectional survey
Source: Updates Surg. 2021 Apr 10;73(5):1983–8. doi: 10.1007/s13304-021-01017-3 (PMC8500901; doi:10.1007/s13304-021-01017-3)
Supplement: Supplementary file 1 — Supplementary file1 (DOCX 23 KB) [file 13304_2021_1017_MOESM1_ESM.docx]

| N° | Type | Question | Answers |
| --- | --- | --- | --- |
| 1 | Redaction | What is your e-mail ? | (e-mail address) |
| 2 | One choice | What is your gender ? | - Male  - Female |
| 3 | Redaction | How old are you? | (years) |
| 4 | Redaction | How many years of surgical experience have you acquired after obtaining your FMH degree in general surgery? | (years) |
| 5 | Multiple choice | What is your surgical qualification? | - No FMH qualification yet  - FMH specialist in general surgery  - FMH specialist in visceral surgery  - European board certification in a subspecialty of visceral surgery |
| 6 | One choice | What is your main field of surgical practice? | - General surgery  - Upper GI surgery (incl. bariatric surgery)  - Lower GI surgery (colorectal surgery)  - Hepatobiliary surgery  - Transplantation  - Other(s) |
| 7 | One choice | What type of institution are you mostly working in? | - University hospital  - Regional hospital  - Private clinic |
| 8 | One choice | When have you first heard about pNPWT (applied on a closed skin)? | - More than 5 years ago  - More than 4 years ago  - More than 3 years ago  - More than 2 years ago  - More than 1 year ago  - More than 6 months ago  - Less than 6 months ago  - I have not heard of pNPWT before the present survey (stops the survey) |
| 9 | One choice | Have you already used a pNPWT system (applied on a closed skin) in your surgical practice ? | - Yes  - No  - Yes, but I have stopped using it (redirection to question 22) |
| 10 | One choice | When was pNPWT first introduced in your Division ? | - More than 5 years ago  - More than 4 years ago  - More than 3 years ago  - More than 2 years ago  - More than 1 year ago  - More than 6 months ago  - Less than 6 months ago |
| 11 | Multiple choice | What pNPWT system(s) is (are) available in your hospital ? | - PREVENA Incision management system (KCI, Acelity)  - PICO Single use negative pressure wound therapy system (Smith and Nephew)  - A system I customize from V.A.C. system (KCI, Acelity)  - Another pNPWT system (redirection to question 11) |
| 12 | Optional redaction | What other pNPWT system(s) are available in your hospital ? |  |
| 13 | Multiple choice | What pNPWT system(s) have you used ? | - PREVENA Incision management system (KCI, Acelity)  - PICO Single use negative pressure wound therapy system (Smith and Nephew)  - A system I customize from V.A.C. system (KCI, Acelity)  - Another pNPWT system (redirection to question 14) |
| 14 | Optional redaction | What other pNPWT system(S) have you used ? |  |
| 15 | Multiple choice | On what type of abdominal wound(s) have you used pNPWT ? | - Midline incision (median laparotomy)  - Subcostal incision  - Mc Burney incision  - Pfannenstiel incision  - Groin incision  - Perineal incision  - Closed stoma wound after stoma closure  - Other(s) |
| 16 | One choice | On what proportion of your patients do you used pNPWT after skin closure ? | - <10%  - 10-25%  - 25-50%  - 50-75%  - 75-100% |
| 17 | One choice | Here is a list of risk factors for SSI : emergency laparotomy, colorectal surgery, diabetes, obesity, thickness of subcutaneous tissue, contaminated wound, immunosuppression. How many of these risk factors do you consider necessary for you to apply pNPWT ? | - 0 (I apply it on all patients with laparotomy)  - 1  - 2  - 3  - 4  - >4 |
| 18 | Multiple choice | According to you, pNPWT allows to prevent ? | - Surgical site infection  - Wound dehiscence  - Incisional hernia  - Bad aesthetic aspect of the scar  - None of the above |
| 19 | One choice | According to you, which of the pNPWT devices you think is the most efficient ? | - PREVENA Incision management system (KCI, Acelity)  - PICO Single use negative pressure wound therapy system (Smith and Nephew)  - A system I customize from V.A.C. system (KCI, Acelity)  - Another pNPWT system |
| 20 | Optional redaction | What are, according to you, the major issues encountered with pNPWT ? |  |
| 21 | Optional redaction | Have you already  What are, according to you, improvements that could be brought to the existing pNPWT systems ? |  |
| 22 | Optional redaction | What was your main reason for discontinuing using pNPWT ? |  |
